# Tuberculose tibiale chez l’enfant à l’IRM: à propos d’un cas

**DOI:** 10.11604/pamj.2019.32.21.17644

**Published:** 2019-01-15

**Authors:** Lynda Nadine Gui Bilé, N’Dri Simon Dédé, Raïssa Michelle Kabas, Estelle Valérie Ohui-Acko, Eric Kouadio, Lolo Marc-Anicet Diambra

**Affiliations:** 1 Service de Radiodiagnostic et d’Imagerie Médicale, CHU de Treichville Côte d’Ivoire/01 BP V3 Abidjan, Côte d’Ivoire

**Keywords:** Tuberculose, os, tibia, IRM, Tuberculosis, bone, tibia, MRI

## Abstract

La localisation osseuse isolée de la tuberculose est peu fréquente et survient surtout chez les adultes jeunes immunodéprimés. Elle peut simuler une infection à germe banal au début ou une atteinte tumorale lorsque l’évolution est chronique. Les auteurs rapportent le cas d'une patiente de 15 ans sans antécédent particulier ayant présenté une tuméfaction indurée et indolore du tiers supérieur de la jambe d'évolution chronique. Les explorations radiologiques conventionnelles ont permis d'objectiver une lésion lytique tibiale métaphysaire supérieure avec une réaction périostée. L’IRM a précisé une rupture corticale et une extension aux parties molles sous la forme d'une collection liquidienne avec une paroi épaisse, se rehaussant intensément après injection de gadolinium. Une biopsie chirurgicale avec analyse histologique a permis de retenir le diagnostic d’ostéite tuberculeuse. Ce cas clinique souligne l'intérêt de l’IRM dans l'exploration des pathologies musculosquelettiques.

## Abstract (en)

Isolated bone involvement of tuberculosis is uncommon and mainly occurs among immunocompromised young adults. Initially, it can mimick an infection due to common germ or a tumor when its evolution is chronic. We here report the case of a 15-year old female patient with no particular previous history presenting with hard and painless swelling on the upper third of the leg with chronic evolution. Common X-ray showed lytic lesion in the upper metaphysiary region of the tibia with periosteal reaction. MRI showed cortical rupture with extension to the soft parts appearing as fluid collection with a thick wall, intensely enhancing after gadolinium injection. Surgical biopsy with histological analysis allowed to retain the diagnosis of tuberculous osteitis. This clinical case emphasizes the role of MRI in the examination of musculoskeletal pathologies.

## Introduction

La tuberculose osseuse est la troisième manifestation de la tuberculose extra pulmonaire. Elle touche préférentiellement la colonne vertébrale (50%), les hanches et les genoux (15%) [1]. Malgré l'état d'endémicité de notre pays, la localisation osseuse isolée est peu fréquente et est surtout observée chez les adultes immunodéprimés. L'objectif de ce travail était de décrire l'apport de l'IRM dans le diagnostic de l'ostéite tuberculeuse à travers une observation faite chez une adolescente.

## Patient et observation

**Figure 1:**
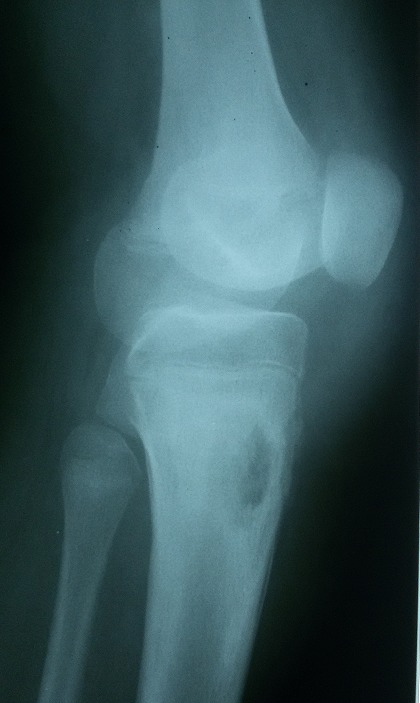
Radiographie du genou. Mise en évidence d'une ostéolyse géographique métaphysaire à limite flou de type Ic de lodwick avec une rupture corticale et une réaction périostée compacte

**Figure 2:**
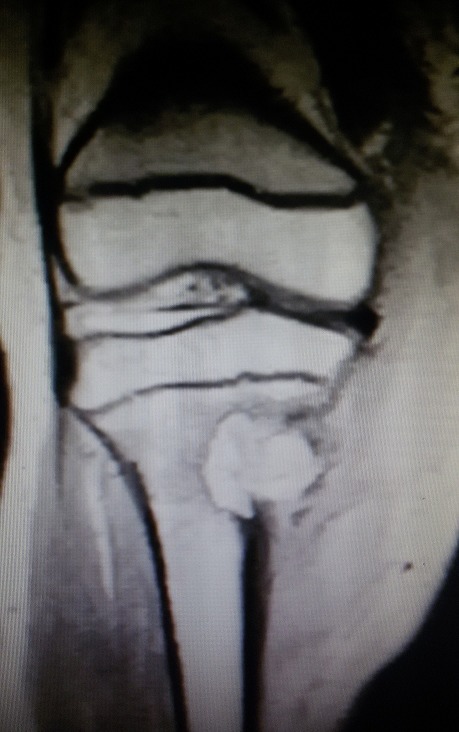
Coupe coronale du genou en EST1 montrant une érosion de la corticale médiale de la métaphyse supérieure du tibia avec une lésion intra-osseuse hétérogène. Présence d'une reconstruction ostéosclérosante délimitant la lésion

**Figure 3:**
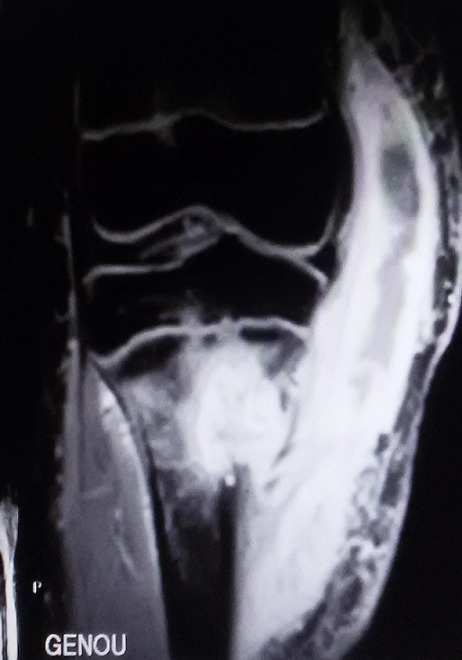
IRM du genou après saturation de graisse et injection de gadolinium (WFS T1 GADO). Mise en évidence de la lésion métaphysaire qui se rehausse intensément avec une atteinte débutante du cartilage de croissance en faveur d'un abcès intra-osseux

**Figure 4:**
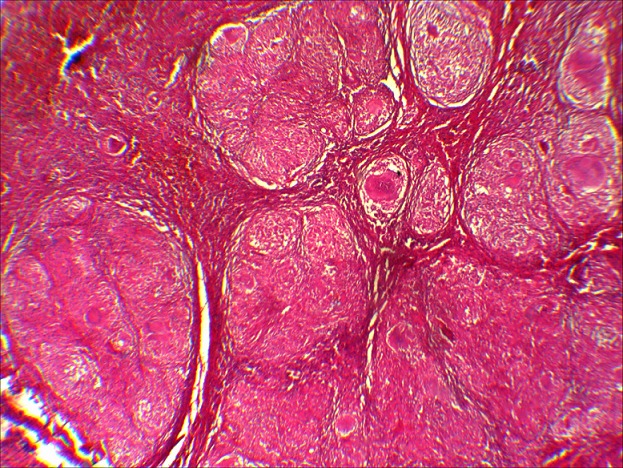
Coupe histologique (hex100). Présence d’un granulome épithélioïde et giganto-folliculaire

B.R. âgée de 15 ans, sans antécédent particulier (BCG fait à la naissance) a été admise dans le service de chirurgie pédiatrique de notre hôpital pour l'exploration d'une tuméfaction indurée du tiers supérieur de la jambe droite évoluant depuis environs 09 mois. Cette masse a été traitée à l'origine comme une ostéomyélite compte tenu du syndrome inflammatoire clinique et biologique fruste observé (discrète hyperleucocytose à polynucléaire neutrophile et augmentation de la CRP). Les premières explorations radiologiques étaient normales, et la patiente ne présentait pas de signe d'imprégnation tuberculeuse. Cependant, les antibiothérapies successives administrées n'ont pas amélioré les signes. A 09 mois d'évolution, la patiente était apyrétique et présentait une importante tuméfaction indolore de la jambe. Des explorations radiologiques ont donc été entreprises, notamment une incidence standard et une IRM du genou droit, toutes deux réalisées le même jour. La radiographie du genou a mis en évidence une ostéolyse géographique de type 1C de la classification de Lodwick au niveau de la métaphyse tibiale sans atteinte du cartilage de croissance avec une rupture de la corticale et une réaction périostée (Figure 1). L'IRM a objectivé sur la séquence morphologique en EST1, une lyse osseuse métaphysaire avec reconstruction sclérosante (Figure 2). Sur les séquences après injection de gadolinium, il est mis en évidence une prise de contraste intense et hétérogène de l'os spongieux métaphysaire tibial avec une rupture de la corticale et une extension aux parties molles sous la forme d'une collection liquidienne (Figure 3). Il n'avait pas d'atteinte de l'articulation du genou. Cet aspect pouvait être en faveur d'une ostéite chronique à germe spécifique. Il ne fallait toutefois pas exclure un processus tumoral avec une infiltration nécrotique des parties molles. Une biopsie chirurgicale a donc été réalisée suivie d'une analyse histologique. Elle confirmait le diagnostic d'ostéite tuberculeuse du tibia avec abcès froid des parties molles, par la mise en évidence de granulome à cellules géantes avec nécrose caséeuse (Figure 4). L'évolution a été favorable sous traitement antituberculeux.

## Discussion

L’ostéite tuberculeuse est habituellement une ostéite chronique avec un long délai diagnostic [2]. Elle se manifeste par des douleurs et/ou une tuméfaction évoluant lentement vers l’aggravation. Le diagnostic reste difficile en l'absence d'une arthrite associée ou une lésion pulmonaire ou extra pulmonaire évocatrice de tuberculose. Et ce d’autant plus que d'autres affections peuvent avoir le même aspect clinique et radiologique comme une tumeur maligne, un abcès de Brodie, ou une hémopathie maligne [3]. Lorsqu’une lésion osseuse isolée existe, elle atteint plus fréquemment le fémur et le tibia [4, 5]. Cependant des localisations plus rares: fibulaire [6], patellaire [7], radiale et ulnaire [4] et métacarpienne [8] ont été rapportées dans la littérature. Et l’imagerie joue un rôle important dans la description des lésions osseuses et des parties molles surtout l’IRM par son excellente résolution de contraste. Cet apport de l’IRM dans le diagnostic de la tuberculose osseuse est peu rapporté [9-11]. Devant le retard radio-clinique des incidences standards, l'IRM permet dans les 48h de mettre en évidence un œdème osseux médullaire par un hyper signal sur la séquence STIR (Short time inversion recuperation). Chez notre patiente, vue en phase chronique, l'IRM a permis de mettre en évidence des lésions aussi bien osseuses, médullaires, cartilagineuses que des parties molles. En effet, l'IRM a objectivé la rupture corticale métaphysaire avec la reconstruction ostéosclérosante qui n'est pas présente dans les lésions tumorales malignes. L'atteinte médullaire et l'extension cartilagineuse sont visibles après injection de produit de contraste. En phase subaiguë, un abcès intra-osseux de granulome est visible sous la forme d'un hypo signal T1 [12]. En T2, il est en hypo signal ou en signal intermédiaire ou d'aspect mixte en anneau. Son rehaussement est variable, homogène ou hétérogène, parfois annulaire. Lœdème médullaire périphérique apparait alors en hypo signal en T1 et en hyper signal T2. Le séquestre osseux est mis en évidence par un hypo signal T1 et T2. L’abcès des parties molles non identifié à la radiographie standard chez notre patiente se présentait à l'IRM avec une paroi épaisse et une régularité des contours malgré l’étendue de la lésion en faveur d'une atteinte tuberculeuse [12]. L'IRM est en outre contributive dans la surveillance des lésions. Le diagnostic de certitude sera posé par l'histologie et le traitement après curetage chirurgical a été favorable sous poly chimiothérapie antituberculeuse.

## Conclusion

Le diagnostic de tuberculose osseuse reste difficile. Certaines localisations bien que peu fréquentes doivent toujours faire évoquer la tuberculose en particulier dans les pays endémiques. Devant des lésions osseuses lytiques d'évolution insidieuse associées ou non à des collections liquidiennes, l’IRM occupe une place essentielle pour un bilan lésionnel et d’extension plus précis. La réalisation de prélèvements avec une étude histologique permettra de confirmer le diagnostic.

## Conflits d’intérêts

Les auteurs ne déclarent aucun conflit d'intérêts.
